# Socio-economic factors affecting quality of life of Hemodialysis patients and its effects on mortality

**DOI:** 10.12669/pjms.344.15284

**Published:** 2018

**Authors:** Muhammad Anees, Shazia Batool, Marium Imtiaz, Muhammad Ibrahim

**Affiliations:** 1 Prof. Dr. Muhammad Anees, Department of Nephrology, King Edward Medical University, Lahore, Pakistan; 2 Ms. Shazia Batool, Department of Nephrology, King Edward Medical University, Lahore, Pakistan; 3 Ms. Marium Imtiaz, Department of Nephrology, King Edward Medical University, Lahore, Pakistan; 4 Mr. Muhammad Ibrahim, Department of Statistics, Muhammadan Anglo Oriental College, Lahore, Pakistan

**Keywords:** Economical factor, Hemodialysis, Mortality, QOL

## Abstract

**Objective:**

Many factors affect quality of life (QOL) of dialysis patients. This study was conducted to determine the effect of socio-economic factors effecting QOL of hemodialysis patients.

**Methods:**

This descriptive multi-centric, follow up study was conducted at Department of Nephrology, Mayo Hospital, Lahore, from February 2015 to August 2017. All patients who were on regular maintenance hemodialysis (MHD) for more than three months and able to read and understand Urdu version of Kidney Disease Quality Of Life (KDQOL) tool were included in the study. Patients were included from hemodialysis units of Mayo Hospital (MH), Shalamar Hospital (SH), and Shaikh Zayed hospital (SZH), Lahore. Patients with less than three-month duration on dialysis, with cognitive impairment, dementia, active psychosis, non-Urdu readers/speakers were excluded. Demographic data and lab data was collected on predesigned pro forma. Patients were divided into different groups on the basis of education, monthly income, source of funding for treatment and employment. Patients were followed up for two years to determine the effect of QOL on mortality.

**Results:**

One hundred and thirty-five patients were included in the study. Socio-economic factors like education, employment, income, funding was compared with KDQOL sub scales and were found statistically significant (p-value (<0.05). We found that patients with higher income had better work status (p=0.039) but social (0.04) and sexual function (p=0.029) were relatively better in patients with low income. Employed patients had better work status (p=0.01), ability to do social function (p=0.027) but they had more pain (0.049), symptoms/problems of disease (p=0.05) and effect of kidney disease (p=0.015). Those patients whose dialysis were funded by their family could socially interact (p=0.012) better and deal more efficiently with effect of kidney disease (p=0.007). Higher education was associated with better emotional well being (p=0.045), patient satisfaction (p=0.046) and staff encouragement (p=0.045) then patient with lower level of education. QOL had no effect on mortality.

**Conclusion:**

The socio-economic factors consisting of education, employment, income and funding are important parameters affecting QOL of kidney patients. QOL does not affect mortality of the dialysis patients.

## INTRODUCTION

As patients develop End Stage Renal Disease (ESRD), life cannot be sustained without renal replacement therapy, which is very expensive. In Pakistan, round about 2.0 Gross Domestic Product (GDP) of the annual budget is spent on health^1^,^2^ which is much less as compared to developed countries.^3^ Similar is the situation in our neighboring countries like India, Sri Lanka, and Iran.^4^,^5^ The major chunk of the money spent on health in Pakistan is on preventive programs of infectious disease (tuberculosis, cholera, typhoid, gastro, malaria and dengue) and mother-child health as this is still the major reason of mortality. Only minor amount of the budget is spent on chronic diseases like diabetes, hypertension, chronic kidney diseases (CKD) and maintenance hemodialysis (MHD). In Punjab, the major province of Pakistan, government is providing free of cost dialysis at all public sector hospitals. These hospitals cater only small number (about 30-40%) of the patients.^6^ Most of the remaining patients either get treatment from their own pocket or being sponsored by different welfare or non-government organizations (NGOs). Most of the international and national data focuses on the medical related factors affecting QOL of dialysis patients. But there is very limited data on the socio-economic factors affecting QOL of dialysis patients and its effect on mortality. So this study was conducted to emphasize its importance.

## METHODS

One hundred and thirty-five patients were included in this study (10 patients were from MH, 35 from SH and 90 from SZH). Patients suffering from ESRD, on MHD for ≥3 months and literate were included in the study. Patients were excluded if they were having cognitive impairment, dementia, non-Urdu readers, and patients on dialysis with less than three-month duration of dialysis. After taking permission from in charge of respective dialysis unit, informed consent was taken from the patients. Demographic data including age, gender, marital status, education, monthly income, employment and lab data was collected on pre designed pro forma. We used kidney disease quality of life (KDQOL) tool for assessing QOL of dialysis patients. The KDQOL instrument is a self-reported measure developed for individuals with kidney disease and on dialysis.^7^ The KDQOL subscale comprise of symptoms, effect of kidney disease, burden of kidney disease, work status, cognitive function, quality of social interaction, sexual function, sleep, social support, dialysis staff encouragement, physical function, role-physical, pain, general health perception, emotional well-being, role emotional, role-emotional, social function, energy/fatigue. Patients under study were provided with translated and validated, Urdu version of KDQOL tool.^8^ Patients were divided into three groups on the basis of monthly income i.e. less than Rs.5000/-, 5000-25000/- and more than 25000/-. Patients were divided into two education groups less than or more than 10 years of education. Expenditure on dialysis led to division of patients into four groups like self-supporting, family support, public hospitals support and sponsored by the serving department where employed. Patients were followed up for two years for mortality.

### Statistical analysis

The data was analyzed using SPSS ver. 20. Continuous variable was expressed as mean ± SD whereas categorical variable was expressed in the form of frequency. One-way ANOVA and T-test was used for comparison of parameters. Chi-Square test was used to observe any association between categorical variable. A p-value less than 0.05 was taken as statistical significant.

## RESULTS

One hundred and thirty-five patients were included in the study. Majority of the patients were unemployed 98 (74.9%), with education >10 years 110(84.6%), middle income class Rs.5000-25000/-month 99 (76.2%) and funded by government 85 (65.4%). The socioeconomic factors like education, employment, income, funding for dialysis was compared with KDQOL sub scales and were found statistically significant (p-value <0.05). We found that patients with more income had better work status (p=0.039) but social (0.04) and sexual function (p=0.029) were relatively better in patients with lowest income status. Employed patients had better work status (p=0.000), ability to do social function (p=0.027) but they had more pain (0.049), symptoms/problems of disease (p=0.05) and limitations that effect (p=0.015) life of patients. Those patients whose dialysis were funded by their family could socially interact (p=0.012) better and deal more efficiently with limitation of disease that effect QOL (p=0.007) while patients whose dialysis were sponsored by their department were having more effect of kidney disease (p=0.007). Higher education was associated with better emotional wellbeing (p=0.045), patient satisfaction (p=0.046) and staff encouragement (p=0.045) then patient with lower level education. QOL had no statistically significant effect on mortality.

## DISCUSSION

Finance is the very important determinant affecting personal, social and health related factors. As Pakistan is a developing country and according to Human Development Index 60.3% of Pakistan’s population live under $1 a day.^9^ This poverty affects not only education, living standard but also leads to life threatening complication of health. In this study average QOL of our patients is similar to neighboring countries like Saudi Arabia^10^, Egypt^11^ and Iran^12^ but poor than developed country like Singapore^13^ (Table-I).

**Table-I:** Showing HRQOL of present study and international studies.

S. No	Sub-scale	Present study (Pakistan)	Al jumaih et al.^10^ (Saudi Arabia)	Veena et al. (Singapore)^13^	Abd el Hafeez et al.^11^(Egypt)	Pakpour AH et al.^12^(Iran)
1	Symptom	78.51(14.36)	77.3(16.3)	-	72.5(11.5)	53.7(24.2)
2	Effect	68.10(21.01)	73(33.5)	-	73.84(13.6)	30.8(21.8)
3	Burden	35.96(25.88)	51(30.7)	-	40.13(26.6)	31.7(25.1)
4	Work status	29.23(33.40)	24.5(35.2)	-	49.0(38.3)	20(30.9)
5	Cognitive function	31.78(36.05)	25.6(9.5)	-	68.73(13.7	55.7(17.3)
6	Social interaction	33.38(23.16)	58.9(29.1)	-	71.40(10.4)	61.7(18.2)
7	Sexual function	70.14(25.51)	81.2(23.3)	-	61.5(23.1)	37.5(34.3)
8	Sleep	63.64(25.49)	66.8(24.4)	-	58.38(15.9)	50.4(18.8)
9	Social support	82.56(23.47)	78.3(29.8)	-	63.17(28.9)	76.8(22.1)
10	Staff encouragement	89.90(16.48)	81.5(26.1)	-	-	68.7(23.1)
11	Pt satisfaction	61.43(11.07)	81.5(26.1)	-	65.67(17.9)	59.7(24.6)
12	Physical functioning	49.0(25.56)	50.4(29.1)	71.47(42.25)	49.1(27.3)	36.9(26.9)
13	Role-physical	26.73(36.59)	35.0(38.8)	59.94(24.15)	28.5(32.0)	22.2(36.3)
14	Pain	52.23(26.78)	61.3(34.8)	77.28(22.79)	44.65(23.1)	42.8(27.5)
15	General Health	49.81(19.48)	58.2(25.0)	50.20(19.05)	37.5(19.0)	40.1(11.0)
16	Emotional well-being	62.65(22.9)	63.7(26.8)	71.53(15.65)	60.84(10.0)	-
17	Role-emotional	31.79(35.91)	37.5(44.6)	78.62(38.20)	63.67(41.9)	27.0(21.1)
18	Social function	58.37(25.88)	58.9(29.1)	69.48(24.14)	54.50(23.4)	53.75(22.7)
19	Energy/fatigue	43.15(22.64)	56.5(28.9)	58.86(17.71)	47.80(14.5)	-

There are many reasons for difference in QOL of our patients. In our system, patients are being referred to nephrologist at very late stage^14^ and they don’t have access for dialysis. On the other side the patients have false myths about dialysis and they think that dialysis means death, leading to refusal of dialysis^15^ on first presentation for dialysis. Later on these patients present to Nephrologist in a very critical condition leading to increased morbidity and mortality of the patients. Initiation of dialysis through the temporary catheter instead of Arterio Venous Fistula leads to line related sepsis and life threatening complications.^16^ In Pakistan there is limited number of public sectors hospitals, that are offering free dialysis but due to limited number of slots most of them offer twice weekly dialysis that is inadequate.^17^ This saves the patient’s life but does not give good quality of life.^18^

QOL is an important parameter of HD patients and is strong predictor of morbidity^19^ and mortality but our result does not correlate with that and poor quality of life does not affect mortality of these patients. In our point of view perhaps medical factors are important and not properly managed leading to high morbidity and mortality which supervene QOL factor. In our study most of the patients were anemic, malnourished and getting inadequate dialysis which was important predictor of morbidity and mortality. So there is need to compare medical and socio economic factors comparing the effect of these factors with morbidity and mortality then final conclusion can be withdrawn for these important observations. In this study high mortality was in patients with more age as compared to young patients. So mortality was naturally due to aging and additional co-morbidities.

In this study, patients with higher income have better QOL in dialysis patients. Actually patients with better economic status have an access to treatment modalities for kidney patients like dialysis and transplants. These patients can afford very expensive treatment in the form of dialysis which costs approx. $30-40/ session. These patients can also afford very expensive medications and nutritious food which help in maintaining their hemoglobin,^20^ control renal osteodystrophy and metabolic profile.^21^ These patients get adequate dialysis which affects morbidity and mortality of the patients. In this study patients with low income have better social interaction and sexual function than patients with higher income group. Actually poor patients have higher number of family members which helps them to interact with each other and share their worries that improves QOL of the patients. Poor patients have less opportunity of recreation, sports and enjoyment activities which are expensive and unsociable so the only way of recreation is sexual enjoyment for them, improving QOL.

**Table-II:** Showing factors affecting QOL of dialysis patients.

S. No	Demographic factors	Subgroups N (%)	Sub scale being Effected	Mean ± SD	P-Value
1	Education	≤10 years 110(84.6) <10 years 20(15.4)	Emotional well-being	64.36±22.972 53.20±20.606	0.045^*^
			Pt. satisfaction	62.26±10.300 56.90±14.057	0.046*
			Staff encouragement	91.14±15.365 83.13±20.788	0.045*
2	Employment	Employed 32(24.6) Unemployed75(57.7) Housewife23(17.7)	Work status	54.69±34.453 17.33±27.867 32.61±28.638	0.000^***^
			Pain	62.27±20.541 48.70±27.074 49.78±30.812	0.049^*^
			Social function	68.36±25.595 53.83±23.874 59.24±29.488	0.027^*^
			Quality of social interaction	24.38±18.128 37.24±23.406 33.33±25.898	0.030^*^
			Symptom/problems	83.53±10.623 77.38±15.162 75.17±14.974	0.05^*^
			Effects of kidney disease	74.41±17.863 63.58±21.223 74.05±21.433	0.015^*^
3	Income	Rs.≤5000 Rs. (3.8%) 5000-25000 Rs. (76.2%) >25000 Rs. (20%)	Work status	20.00±27.386 26.26±32.999 40.00±32.275	0.039^*^
			Social function	82.50±20.917 55.93±24.507 61.50±29.074	0.040^*^
			Sexual function	100.00±0.00 66.11±25.509 89.06±14.075	0.029*
4	Funding	Self-17 (13.1) Family support 17(13.1) GOVT 85(65.4) Other11(8.5)	Quality of social interaction	21.96±18.068 42.35±18.700 35.53±23.869 20.61±21.79	0.012^*^
			Effect of kidney disease	71.69±16.809 55.33±19.097 68.09±21.705 82.39±13.352	0.007^*^

In this study employment is important factor affecting QOL of the HD patients. Employed patients are independent and earning for themselves which makes them confident and secure in getting treatment. Employed patients have daily routine activity of going to office and spent time there with their colleagues which keep them busy and socially active^22^ improving their QOL. But employed patients have more pain and symptoms of disease than unemployed patients because of remaining busy throughout the day. Whereas unemployed patients have less pain, symptoms of the disease and better social function as they have nothing to do and remain free throughout the day which allows them to have interaction with the family members, relatives and friends.

In this study we found that patients whose dialysis were funded by their family have good quality of social interaction and less effected by fluid restriction and dietary restriction than those patients who had dialysis at government/NGO funded dialysis centers. Patients had better ability to do household and ability to travel. Patients were less dependent on doctors or staff. They were less stressed, had good sex life and were less worried about their personal appearance. Patients who were supported by their family were less affected because of care given by family.^23^ Patients whose dialysis was done by their sponsored organization had more effect of kidney disease because they have to work for organization which affects them.

In this study, education was another important factor affecting QOL of the HD patients. Educated patients have better understanding of the disease and its treatment modalities. They can understand medical terms easily. That’s why staff can guide educated patients more easily about his diet, medication, its effects and side effects, fluid & salt restrictions and disease influence on his future life. Educated patients have an access to health related soft and hard material from which they gain guidance. If educated patients are counseled about the disease they accept it which improves quality of life.^24^ People with low education level have lower quality of life because they can’t understand what actually happens through replacement therapies and why dialysis staff is restricting eatables so they become frustrated which effect their emotional well-being and make them more dissatisfied. That’s why they often remained malnourished and in fluid overload.^25^ Similar results were observed by other studies. In this study it was seen that morbidity and mortality had no effect on quality of life of dialysis patients.

## CONCLUSION

The socio-economic factors consisting of education, employment, income and funding are important parameters affecting QOL of kidney patients. QOL does not affect mortality of the dialysis patients.

### Authors’ Contribution

**MA:** The conception and design of the study, final approval of manuscript.

**MI and SB:** Acquisition of data, drafting the article.

**MI:** Analysis and interpretation of data.
